# Manganese Exposure in Occupational Settings: Disruptions in Endothelial Function and Thyroid Regulation

**DOI:** 10.3390/metabo16010001

**Published:** 2025-12-19

**Authors:** Melih Gaffar Gözükara, Servet Birgin İritaş, Lütfiye Tutkun, Murat Büyükşekerci, Özlem İritaş, Vugar Ali Türksoy, Deniz Özkan Vardar, Serdar Deniz, Engin Tutkun

**Affiliations:** 1 Department of Public Health, Faculty of Medicine, Ankara Yildirim Beyazit University, Ankara 06000, Türkiye; mggozukara@aybu.edu.tr; 2 Department of Forensic Medicine, Faculty of Medicine, Ankara Yildirim Beyazit University, Ankara 06000, Türkiye; 3 The Association of Industrial Toxicology and Occupational Hygiene, Ankara 06660, Türkiye; lutfiye.tutkun@etok-ihider.org; 4 Department of Pharmacology, Ankara Ataturk Sanatorium Training and Research Hospital, Ankara 06290, Türkiye; murat.buyuksekerci@saglik.gov.tr; 5 Republic of Türkiye Ministry of Agriculture and Forestry, General Directorate of State Hydraulic Works, Ankara 06800, Türkiye; ozlemiritas@dsi.gov.tr; 6 Department of Public Health, Faculty of Medicine, Yozgat Bozok University, Yozgat 66900, Türkiye; v.aliturksoy@bozok.edu.tr; 7 Department of Pharmacy Services, Vocational School of Health Services, Lokman Hekim University, Ankara 06510, Türkiye; deniz.ozkanvardar@lokmanhekim.edu.tr; 8 Department of Public Health, Faculty of Medicine, Malatya Turgut Özal University, Malatya 44900, Türkiye; serdar.deniz@ozal.edu.tr; 9 Tez Medikal Institute of Occupational Health and Safety, İstanbul 34413, Türkiye

**Keywords:** manganese exposure, welding fumes, thyroid hormones, T3, T4, TSH, ADMA, SDMA, endothelial dysfunction, metabolomics

## Abstract

**Background:** Manganese (Mn) exposure is common in welding and metal-processing occupations and has been implicated in both thyroid disruption and endothelial dysfunction through oxidative and nitric-oxide–related pathways. However, endocrine and vascular biomarkers have rarely been examined together in occupational settings. **Methods:** In this cross-sectional study, 95 Mn-exposed workers and 95 non-exposed controls were evaluated. Whole-blood Mn, triiodothyronine (T3), thyroxine (T4), thyroid-stimulating hormone (TSH), asymmetric dimethylarginine (ADMA), symmetric dimethylarginine (SDMA), arginine and citrulline were measured using validated Inductively Coupled Plasma—Mass Spectrometer and chemiluminescent immunoassays. Group differences were assessed using independent samples t-tests, and exposure–biomarker associations were evaluated using Pearson correlations (*p* < 0.05). **Results:** Mn-exposed workers had significantly higher blood Mn levels than controls (19.82 ± 4.54 vs. 10.22 ± 3.07 µg/L; *p* < 0.001). Thyroid hormones (T3, T4, and TSH) were significantly lower among Mn workers, representing a non-classical hormonal pattern, including T3 (2.47 ± 0.31 vs. 3.14 ± 0.42 ng/L; *p* < 0.001), T4 (1.02 ± 0.13 vs. 1.21 ± 0.18 ng/L; *p* < 0.001), and TSH (1.75 ± 0.53 vs. 2.88 ± 0.37 mIU/L; *p* < 0.001). Endothelial biomarkers also differed: ADMA (0.26 ± 0.14 vs. 0.19 ± 0.08 µmol/L; *p* < 0.001) and SDMA (0.24 ± 0.06 vs. 0.20 ± 0.03 µmol/L; *p* < 0.001) were higher, while citrulline was lower (18.77 ± 10.23 vs. 22.82 ± 6.70 µmol/L; *p* = 0.002). In Mn workers, blood Mn showed negative correlations with T3 (r = –0.535, *p* < 0.01), T4 (r = –0.331, *p* < 0.01), and TSH (r = –0.652, *p* < 0.01), and positive correlations with ADMA (r = 0.205, *p* < 0.05) and SDMA (r = 0.193, *p* < 0.05). **Conclusions:** These findings indicate measurable differences in thyroid hormones and dimethylarginine-related endothelial markers among Mn-exposed workers. While the cross-sectional design precludes causal inference, the combined pattern suggests a possible unusual biological response involving both endocrine regulation and nitric-oxide–related pathways. Further longitudinal studies incorporating oxidative stress markers, co-exposure assessment, and functional endothelial testing are needed to clarify the biological relevance of these associations.

## 1. Introduction

Manganese (Mn) is an essential trace element involved in mitochondrial antioxidant defense, amino acid metabolism, and endocrine regulation. Nevertheless, chronic exposure to elevated Mn concentrations—frequently encountered in welding, metal processing, and alloy production—has been linked to neurotoxicity, oxidative stress, mitochondrial dysfunction, and systemic metabolic alterations [1,2,3,4]. Welding fumes constitute a complex aerosol of ultrafine metal oxides, and Mn is one of the most abundant toxicologically relevant components. Importantly, welders are exposed to Mn, which affects endocrine or vascular pathways [5,6]. This complexity has contributed to inconsistent findings in occupational studies and highlights the need for biomonitoring-based analyses in well-defined worker populations.

Growing evidence suggests that Mn may influence thyroid hormone homeostasis, although human data remain inconsistent [4,7]. Thyroid hormone synthesis and regulation depend on proper function of deiodinase enzymes, intact oxidative balance, and hypothalamic–pituitary–thyroid (HPT) axis signaling [8]. Mn can disrupt oxidative pathways, alter dopaminergic regulation of thyroid-stimulating hormone (TSH) release, and modulate deiodinase activity in experimental systems [9,10,11]. Some human studies report associations between higher Mn exposure and lower Triiodothyronine (T3) or thyroxine (T4) levels, or altered TSH [12,13], while others report no clear relationship [7]. Differences in exposure characteristics, population heterogeneity, analytical methods, and co-exposure profiles likely contribute to these inconsistencies. These gaps underscore the relevance of examining thyroid hormones in a homogenous cohort of occupationally exposed workers with well-defined internal Mn dose measurements.

Although several experimental and human studies have reported reductions in T3, T4, or altered TSH patterns among Mn-exposed populations [7,9,11,12,13], these findings are not fully consistent across studies because of heterogeneous exposure profiles, differing analytical approaches, and variations in population characteristics. Moreover, despite these repeated observations, earlier research has rarely examined thyroid alterations together with endothelial or nitric oxide (NO) related pathways, even though Mn is mechanistically linked to oxidative stress, mitochondrial disturbance, and vascular dysfunction [1,2,5,14,15] addressing this gap is important for clarifying whether endocrine and vascular responses occur concurrently or represent distinct physiological processes.

Mn has also been implicated in endothelial and vascular disturbances through mechanisms involving oxidative stress, mitochondrial injury, and altered NO signaling. Circulating methylated arginine derivatives provide sensitive indicators of endothelial function: asymmetric dimethylarginine (ADMA) inhibits endothelial NO synthase, while symmetric dimethylarginine (SDMA) competes with arginine for transport and reflects renal and vascular alterations [14]. Elevated ADMA and SDMA levels are associated with hypertension, reduced NO bioavailability, chronic inflammation, and increased cardiovascular risk. Experimental studies demonstrate that Mn can disrupt endothelial integrity and redox homeostasis in blood–brain barrier and vascular endothelial cell models [2,15]. Despite these mechanistic observations, human studies directly linking Mn exposure to ADMA or SDMA alterations remain extremely limited, and no previous occupational study has examined thyroid hormones and methylated arginine profiles together.

Interpreting such biomarker patterns requires attention to potential confounding, including age, smoking, alcohol consumption, renal function, nutrition (iodine, selenium, zinc), physical activity, and concurrent metal exposures [16]. Differences in pre-analytical processing, delays before centrifugation, and analytical batch effects may also influence biomarker stability and comparability. Many prior studies lacked detailed exposure characterization, undermining assessment of dose–response relationships and biological plausibility.

Given these uncertainties, we sought to clarify whether chronic occupational Mn exposure is associated with alterations in thyroid hormone homeostasis and early endothelial dysfunction markers. Based on mechanistic evidence linking Mn to disrupted HPT axis signaling and impaired synthesis and cellular availability/uptake of NO, we anticipated that Mn-exposed workers would show lower T3 and T4 concentrations and altered TSH regulation, as well as higher ADMA and SDMA levels with corresponding reductions in arginine and citrulline. Furthermore, we expected that whole-blood Mn concentrations would correlate with these biomarkers, and that such associations would remain evident when analyses were restricted to the Mn-exposed group alone, thereby reducing confounding by between-group differences. By integrating endocrine and vascular biomarkers within the same occupational cohort, this study aims to provide a clearer understanding of whether chronic Mn exposure contributes to subtle but measurable systemic effects.

## 2. Materials and Methods

### 2.1. Study Design and Population

This cross-sectional study was conducted among male workers employed in metal-related industries with documented occupational exposure to manganese (Mn) and a comparison group of non-exposed men. A total of 190 participants were included: 95 Mn-exposed workers (hereafter “Mn workers”) and 95 comparison subjects frequency matched on age (±3 years).

Welding workers employed in an automotive industrial complex located in the Marmara region, where welding fumes constituted the principal source of Mn exposure. According to workplace inspections and worker reports, welding tasks were typically performed in semi-enclosed areas with variable local ventilation, and the use of respiratory protective equipment was inconsistent across workers. Because personal protective equipment (PPE) use was not systematically documented and differed by task and shift, it could not be quantitatively incorporated into exposure assessment. Eligibility criteria for the exposed group included full-time employment in welding or Mn-related metal work for at least one year and absence of known thyroid disease, chronic kidney disease or cardiovascular disorders.

Comparison subjects were recruited from non-exposed administrative or support staff and from the surrounding community within the same geographic region. Controls were required to have no occupational exposure to welding fumes or Mn-rich environments and to be free of thyroid, renal, or cardiovascular disease. Individuals using medications or supplements known to interfere with thyroid function—such as iodine, selenium, thyroid hormone preparations or glucocorticoids—were excluded. In addition, smoking and regular alcohol consumption were exclusion criteria for both the Mn-exposed workers and the control group. Thus, the same lifestyle-related criteria were applied symmetrically to both groups.

All participants were male and resided in the same geographic area, reducing variability in background environmental exposures, diet, and socioeconomic conditions. Demographic and occupational information, including age, weight, and height, was obtained using a structured questionnaire administered by trained personnel. We did not adjust for duration of exposure in the main analyses because reliable information on the workers’ use of PPE, ventilation conditions, and task-specific exposure variability was not available. Since these factors can substantially modify individual inhalational uptake, self-reported exposure duration alone was not considered an accurate proxy of true cumulative exposure.

### 2.2. Blood Sampling and Processing

Venous blood samples were collected from all participants at the end of the work shift on a typical working day. Whole blood for Mn determination was drawn into trace-metal–free ethylenediaminetetraacetic acid tubes, while blood for thyroid hormones and methylated arginines was collected into serum separator tubes.

Immediately after phlebotomy, samples were gently inverted and stored at 4 °C until processing. Serum tubes were allowed to clot and were centrifuged according to the manufacturer’s instructions. After centrifugation, serum aliquots were transferred into polypropylene tubes and stored at −80 °C until analysis. Whole-blood samples for Mn analysis were similarly stored at −80 °C.

All samples from Mn workers and comparison subjects were handled using the same standard operating procedures and processed in the same laboratory, thereby minimizing systematic differences in pre-analytical handling between groups. The potential impact of processing delays on biomarker stability is discussed as a methodological limitation.

### 2.3. Determination of Whole-Blood Manganese

Preparation of serum samples for Mn analysis was performed using the MARSXpress microwave digestion system (CEM Corporation, Matthews, NC, USA) To each 1 mL serum sample, 5 mL of 65% nitric acid (Merck) and ultrapure water were added and placed in Teflon containers. After digestion, the samples were transferred to polypropylene tubes, and the total volume was increased to 20 mL with deionised water. These samples were stored at +4 °C until analyzed by Inductively Coupled Plasma—Mass Spectrometer (Agilent 7700×, Agilent Technologies, Tokyo, Japan). The calibration curve was constructed using High Purity Standards and showed an excellent linear relationship (r^2^ = 0.9998). The accuracy of the method was confirmed using Seronorm brand Certified Reference Materials (CRM). Mn levels were reported in units of μg/L. The analytical method validation was conducted using CRM (Whole Blood Level 2, Sero AS, Norway). CRMs are highly accurate reference materials certified for one or more specific properties and are crucial for maintaining accuracy and control in analytical processes, ensuring the reliability of results. They are extensively utilized for method validation and optimization, particularly in toxicology. The selected CRM consists of over 95% human blood without any added preservatives or stabilizers, closely mimicking the natural characteristics of human blood. This composition minimizes potential matrix interferences, improving analytical outcomes’ accuracy. The CRM is accompanied by comprehensive documentation, detailing certified analytical values for more than 60 elements, with a primary focus on Mn. These values have undergone independent verification and are traceable to international reference standards, ensuring the highest precision and reliability in analytical measurements. Consequently, CRMs are indispensable for researchers and professionals seeking precise and dependable data. Five replicate measurements were carried out for both standard and sample analyses to verify the accuracy and consistency of our analytical results. During the validation, CRM samples were analyzed five times per day over a period of 10 consecutive days. Both within-day and between-day precision were assessed using Standard Reference Materials (SRMs), with precision determined by calculating the standard deviation of repeated measurements, serving as a quality control indicator. This methodology was implemented to reduce variability, achieving a relative standard deviation of less than 5%. Specifically, for the Mn method validation, the coefficient of variation (CV) for the Whole Blood Level 2 (WBL-2) CRM was determined to be 3.17%.

### 2.4. Measurement of Thyroid Hormones and Methylated Arginines

Serum total triiodothyronine (T3), thyroxine (T4), and TSH were quantified using Siemens IMMULITE 2000 XPi immunoassay system (Siemens Healthcare Diagnostics, Tarrytown, NY, USA) in accordance with the manufacturer protocols. All assays underwent routine internal quality-control procedures, and both intra- and inter-assay coefficients of variation were <5%. The analytical ranges were sufficient to detect normal as well as subclinical hypo- and hyperthyroid states.

Arginine and citrulline levels, together with methylated arginine derivatives (ADMA, SDMA) in serum, were measured by Shimadzu LC-20AD liquid chromatography system (Shimadzu Corporation, Tokyo, Japan) and Applied Biosystems MDS SCIEX API 3200 mass spectrometer (SCIEX, MA, USA) operating in positive electrospray ionization mode. A Phenomenex Luna C18 column was used for separation, and a method described in the literature was modified [14]. At the sample preparation stage, 100 μL of methanol containing d7-ADMA was added to 200 μL of serum. The mixture was centrifuged at 13,000 rpm for 10 min to precipitate proteins. The supernatant was evaporated by drying at 60 °C under nitrogen gas. The residue was dissolved in freshly prepared butanol solution containing 5% volume per volume (*v*/*v*) acetic chloride and incubated at 60 °C for 20 min. This solvent was also evaporated with nitrogen gas. Finally, the derivatised samples were re-dissolved in a volume of 100 μL with a water-methanol mixture (90:10, *v*/*v*) containing 0.1% formic acid, and 40 μL was injected into the ultra-performance liquid chromatography system. Multiple Reaction Monitoring analysis was conducted using ion source cone voltage and collision energy parameters that were optimized during preliminary continuous-infusion tests. The method gave reliable results with CVs of 8.4% and 9.9% for same-day and different-day analyses, respectively.

### 2.5. Statistical Analysis

Statistical analyses were performed using IBM Statistical Package for Social Sciences Statistics version 20.0 (IBM Corporation, Armonk, NY, USA). All continuous variables were analyzed for their distributions by means of the Shapiro–Wilk test for normality and the Levene’s test to analyze the homogeneity of variances. Since each primary variable met these assumptions, only parametric statistical tests were utilized in this study. All continuous variables were summarized as mean ± standard deviation (SD). Group differences between Mn workers and comparison subjects in demographic characteristics, blood Mn, thyroid hormones, and methylated arginines were assessed using independent samples t-tests. Exposure–response relationships between whole-blood Mn and thyroid/methylated arginine biomarkers were evaluated using Pearson or Spearman correlation coefficients, first in the total study population and then restricted to Mn workers alone to reduce between-group heterogeneity. Effect size (Cohen’s d) calculations were performed using G*Power v. 3.1.9.7 (Heinrich Heine University Düsseldorf, Germany).

All tests were two-sided, and a *p*-value < 0.05 was considered statistically significant. Given the exploratory nature of this study, no formal correction for multiple comparisons was applied; however, patterns of association were interpreted cautiously in the context of biological plausibility and consistency.

### 2.6. Ethical Considerations

The study was conducted in accordance with the Declaration of Helsinki. The study protocol was reviewed and approved by the Ankara Bilkent City Hospital Medical Research and Ethics Review Board (Approval No: TABED-1-24-403, Date: 28 August 2024). All participants received written and verbal information about the study and provided written informed consent prior to enrollment.

## 3. Results

A total of 190 male participants were included, with 95 Mn-exposed workers and 95 controls. There were no significant differences between groups in age, body mass index (BMI), hemoglobin, hematocrit, or platelet counts (all *p* > 0.05), indicating comparable baseline characteristics (Table 1).

### 3.1. Blood Manganese Levels

Whole-blood Mn concentrations were significantly higher in the Mn-exposed group compared with the control group (19.82 ± 4.54 µg/L vs. 10.22 ± 3.07 µg/L; *p* < 0.001). This finding demonstrates a clear statistical differentiation between occupationally exposed workers and non-exposed controls and confirms the presence of elevated internal Mn burden among Mn-exposed participants (Table 1).

### 3.2. Thyroid Hormones

Serum concentrations of thyroid hormones differed significantly between Mn-exposed workers and controls. Specifically, Mn-exposed workers exhibited lower levels of T3, T4, and TSH compared with the control group. Mean T3 concentrations were 2.47 ± 0.31 ng/L in the exposed group versus 3.14 ± 0.42 ng/L in controls, while T4 levels were 1.02 ± 0.13 ng/L and 1.21 ± 0.18 ng/L, respectively (both *p* < 0.001). TSH concentrations were also significantly lower among Mn-exposed workers (1.75 ± 0.53 mIU/L vs. 2.88 ± 0.37 mIU/L, *p* < 0.001) (Table 1).

### 3.3. Endothelial Biomarkers

Comparisons of arginine–NO pathway-related biomarkers revealed significant differences between Mn-exposed workers and controls. Serum concentrations of ADMA and SDMA were significantly higher in the exposed group, whereas citrulline concentrations and the arginine/ADMA ratio were significantly lower. ADMA levels were 0.26 ± 0.14 µmol/L in Mn-exposed workers compared with 0.19 ± 0.08 µmol/L in controls, and SDMA levels were 0.24 ± 0.06 µmol/L versus 0.20 ± 0.03 µmol/L, respectively (both *p* < 0.001). Citrulline concentrations were reduced in the exposed group (18.77 ± 10.23 µmol/L vs. 22.82 ± 6.70 µmol/L, *p* = 0.002), accompanied by a lower arginine/ADMA ratio (*p* < 0.001). Although mean arginine levels were lower in Mn-exposed workers, this difference did not reach statistical significance (*p* = 0.072) (Table 1). Serum creatinine levels were comparable between Mn-exposed workers and controls, indicating no relevant difference in excretory renal function between groups which could affect ADMA and SDMA levels (Table 1).

### 3.4. Correlation Analyses

Correlation analyses were performed to evaluate associations between whole-blood Mn concentrations and biochemical parameters within the Mn-exposed group. Whole-blood Mn levels showed significant inverse correlations with thyroid hormones, including T3 (r = −0.535, *p* < 0.01), T4 (r = −0.331, *p* < 0.01), and TSH (r = −0.652, *p* < 0.01). In contrast, Mn concentrations were positively correlated with ADMA (r = 0.205, *p* < 0.05) and SDMA (r = 0.193, *p* < 0.05), while a negative correlation was observed with citrulline (r = −0.229, *p* < 0.01) (Table 2).

Strong inter-correlations were observed among thyroid hormones, as well as between arginine-related metabolites, including a robust correlation between ADMA and SDMA (r = 0.719, *p* < 0.01). These correlation patterns are presented in detail in Table 2.

## 4. Discussion

This study examined thyroid-related and endothelial biomarkers in a group of Mn-exposed workers compared with non-exposed controls. Although the design does not allow causal interpretation, the overall pattern observed—lower thyroid hormone levels and higher dimethylarginine concentrations in Mn-exposed workers—may suggest that chronic exposure could be associated with coordinated changes in endocrine and NO–related pathways. These findings should be interpreted cautiously and viewed as preliminary evidence requiring further confirmation.

### 4.1. Thyroid Hormone Alterations in Manganese Exposure

Workers with higher Mn levels showed lower concentrations of T3, T4, and TSH. Although causality cannot be inferred, previous experimental studies have reported that Mn exposure may influence thyroid hormone synthesis and deiodinase-mediated conversion through oxidative processes [9,11,13,17]. Some human studies have also described inverse associations between Mn and thyroid parameters, including reduced FT4 concentrations in pregnant women [12]. The observed reduction in TSH despite lower peripheral hormones is notable; however, whether this reflects central neuroendocrine modulation remains uncertain. Prior mechanistic work has shown that Mn can accumulate in basal ganglia and interfere with dopaminergic pathways involved in pituitary signaling [1,18,19], though the current study cannot determine whether such pathways contributed to the present findings.

### 4.2. Endothelial Dysfunction Reflected by Altered Methylated Arginines

Mn-exposed workers also exhibited higher serum ADMA and SDMA concentrations and a lower arginine/ADMA ratio. These metabolites are frequently viewed as indirect indicators of altered NO metabolism or early endothelial perturbation, but multiple influences—including oxidative status, renal handling, inflammatory processes, or other co-exposures—may contribute to these differences [14]. Experimental data indicate that Mn can induce oxidative stress and mitochondrial dysfunction in endothelial or neuronal cells [2,15]. Oxidative stress has been shown to inhibit dimethylarginine dimethylaminohydrolase (DDAH), the enzyme responsible for ADMA degradation [20], providing a potential explanation for higher dimethylarginine levels; however, the present cross-sectional data cannot determine whether Mn was directly responsible for such alterations. The modest correlations between Mn and ADMA/SDMA observed here should therefore be interpreted with caution.

Although Mn-exposed workers showed higher ADMA and SDMA levels and lower citrulline concentrations, these effects were noticeably less marked than those observed for thyroid hormones. Both the effect sizes and the statistical robustness of the endothelial associations were weaker, suggesting that alterations in dimethylarginine pathways may represent a subtler or secondary disturbance compared with the more pronounced endocrine changes. Therefore, while endothelial markers showed patterns compatible with NO-related perturbations, the strength of these associations should be interpreted with greater caution.

### 4.3. Potential Shared Mechanisms Linking Endocrine and Endothelial Changes

A plausible biological link between the observed endocrine and endothelial alterations may involve Mn-induced oxidative stress as a shared upstream mechanism. Experimental evidence suggests that Mn can disrupt hypothalamic–pituitary–thyroid (HPT) axis regulation by impairing deiodinase activity and modulating dopaminergic control of TSH secretion [9,10,11,17,18,19]. In parallel, oxidative stress is known to inhibit dimethylarginine dimethylaminohydrolase (DDAH), leading to accumulation of asymmetric dimethylarginine (ADMA) and reduced nitric oxide bioavailability [14,20,21,22].

### 4.4. Implications and Future Directions

The combined alterations observed in thyroid hormones (T3, T4, TSH) and dimethylarginine-related markers (ADMA, SDMA) suggest that chronic Mn exposure may influence multiple physiological pathways, although the magnitude of these effects differed across systems. The stronger and more consistent associations between Mn and thyroid parameters indicate that the HPT axis may be particularly sensitive to Mn-related disturbances, potentially through oxidative mechanisms or altered neuroendocrine regulation. In contrast, the relatively weaker changes in ADMA, SDMA, and citrulline are more compatible with subtle modifications in NO-related pathways rather than overt endothelial dysfunction.

**Figure 1:**
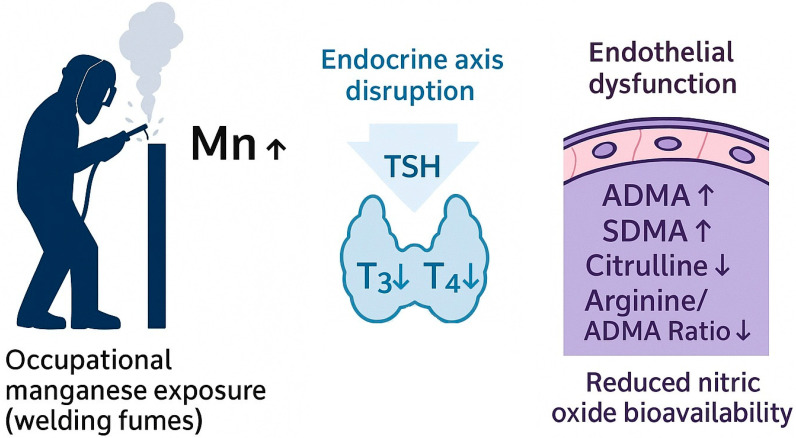
A possible Mn-ADMA-SDMA-Thyroid Alteration Pathway.

Taken together, our findings could point to occupational manganese exposure may induce concurrent disturbances in the hypothalamic–pituitary–thyroid axis and endothelial nitric oxide pathways. A schematic overview of the proposed integrated mechanism is presented in Figure 1.

Oxidative stress remains a plausible shared upstream mechanism linking endocrine and vascular responses to Mn. Experimental evidence shows that Mn can disrupt deiodinase activity and influence dopaminergic regulation of TSH release within the HPT axis, while also impairing NO synthesis through effects on DDAH activity and methylated arginine turnover. The pattern observed in this cohort may therefore reflect parallel but mechanistically distinct processes, with endocrine alterations appearing more pronounced than changes in arginine–NO metabolism.

Further research is needed to clarify the temporal sequence and biological relevance of these biomarker alterations. Longitudinal studies incorporating repeated Mn measurements, assessment of co-exposures, and inclusion of oxidative stress markers or direct measures of deiodinase and DDAH activity would help determine whether these findings represent early adaptive responses, subtle Mn-related perturbations, or interactions with workplace conditions. Such approaches are essential for defining the clinical and mechanistic significance of these observations.

## 5. Conclusions

In this occupational cohort, Mn exposure was associated with measurable alterations in both endocrine- and NO-related pathways, with the most pronounced effects observed in thyroid hormones (T3, T4, TSH). These findings suggest that the HPT axis may exhibit greater sensitivity to chronic Mn exposure compared with dimethylarginine-associated endothelial pathways, where changes were present but less marked in magnitude and statistical consistency.

While the cross-sectional design precludes causal inference, the overall pattern indicates a possible early biological response involving parallel but mechanistically distinct processes. Further longitudinal studies incorporating repeated Mn measurements, oxidative stress markers, and direct assessment of deiodinase and DDAH activity are needed to clarify the temporal dynamics and functional significance of these biomarker alterations.

## Figures and Tables

**Table 1:** Demographic characteristics and biochemical parameters of control group and Mn-exposed group.

Variables with Normal Ranges	Mn-Exposed (n = 95, Mean ± SD)	Control (n = 95, Mean ± SD)	*p* *, es **
Age (year)	41.49 ± 7.74	42.51 ± 8.52	0.393, 0.125
BMI (kg/m^2^) (18.5–24.9)	27.97 ± 2.85	28.14 ± 2.55	0.670, 0.062
Whole blood Mn (μg/L) (4–15)	19.82 ± 4.54	10.22 ± 3.07	<0.001, 2.485
Hemoglobin (g/dL) (13.5–17.2)	14.97 ± 1.04	15.22 ± 0.75	0.055, 0.275
Hematocrit (%) (39.5–50.5)	46.07 ± 2.36	46.41 ± 1.77	0.260, 0.163
Platelet (×10^3^/µL) (150–450)	225.13 ± 33.80	230.00 ± 36.08	0.338, 0.139
T3 (ng/L) (2.3–4.2)	2.47 ± 0.31	3.14 ± 0.42	<0.001, 1.815
T4 (ng/L) (0.89–1.76)	1.02 ± 0.13	1.21 ± 0.18	<0.001, 1.210
TSH (mIU/L) (0.55–4.78)	1.75 ± 0.53	2.88 ± 0.37	<0.001, 2.472
Arginine (µmol/L)	77.44 ± 27.07	88.55 ± 53.27	0.072, 0.263
Citrulline (µmol/L)	18.77 ± 10.23	22.82 ± 6.70	0.002, 0.468
Serum creatinine (µmol/L)	98.18 ± 16.87	91.13 ± 15.22	0.095, 0.438
ADMA (µmol/L)	0.26 ± 0.14	0.19 ± 0.08	<0.001, 0.614
SDMA (µmol/L)	0.24 ± 0.06	0.20 ± 0.03	<0.001, 0.843
Arginine/ADMA	357.25 ± 197.34	493.01 ± 288.60	<0.001, 0.549
SDMA/ADMA	0.99 ± 0.24	1.13 ± 0.21	<0.001, 0.620

* The independent t test was used, ** Effect size (Cohen’s D value), SD: Standard deviation, BMI: Body-mass index, T3: Triiodothyronine, T4: Thyroxine, TSH: Thyroid-stimulating hormone, ADMA: Asymmetric dimethylarginine, SDMA: Symmetric dimethylarginine. Reference ranges represent typical adult male values derived from clinical laboratory standards and published literature. Reference intervals for ADMA, SDMA, arginine, and citrulline may vary depending on analytical method and laboratory.

**Table 2:** Correlations Serum Manganese Levels, Thyroid Hormones (T3, T4, TSH) and Arginine Metabolism Related Compounds in Mn Exposed group.

n = 95	Whole Blood Mn (μg/L)	T3 (ng/L)	T4 (ng/L)	TSH	Arginine	Citrulline	ADMA	SDMA
T3 (ng/L)	−0.535 *	1						
T4 (ng/L)	−0.331 *	0.469 *	1					
TSH (mIU/L)	−0.652 *	0.474 *	0.421 *	1				
Arginine (µmol/L)	−0.045	−0.028	0.147 *	0.198 *	1			
Citrulline (µmol/L)	−0.229 **	0.022	0.138	0.276 *	0.086	1		
ADMA (µmol/L)	0.205 *	−0.533 *	−0.343 *	−0.208 *	−0.09	−0.016	1	
SDMA (µmol/L)	0.193 *	−0.608 *	−0.337 *	−0.232 *	−0.057	−0.053	0.719 *	1
Arginine/ADMA	−0.12	0.268 *	0.260 *	0.291 *	0.857 *	0.01	−0.467 *	−0.416 *

* Correlation is significant at *p* < 0.05, ** *p* < 0.00. T3: Triiodothyronine, T4: Thyroxine, TSH: Thyroid-stimulating hormone, ADMA: Asymmetric dimethylarginine, SDMA: Symmetric dimethylarginine.

## Data Availability

The data presented in this study are available on request from the corresponding author. The data are not publicly available due to privacy or ethical restrictions.

## Abbreviations

The following abbreviations are used in this manuscript:

ADMA	Asymmetric dimethylarginine
BMI	Body mass index
CRM	Certified Reference Materials
CV	Coefficient of variation
DDAH	Dimethylarginine dimethylaminohydrolase
HPT	Hypothalamic–pituitary–thyroid
MN	Manganese
NO	Nitric oxide
PPE	Personal protective equipment
SD	Standard deviation
SDMA	Symmetric dimethylarginine
T3	Triiodothyronine
T4	Thyroxine
TSH	Thyroid-stimulating hormone
*v*/*v*	Volume per volume
